# Phylogenetic relatedness of methicillin-resistant *Staphylococcus aureus* isolates from the host community and Syrian refugees in Duhok Governorate based on *16S rRNA*

**DOI:** 10.1016/j.ijregi.2022.06.003

**Published:** 2022-06-06

**Authors:** Narin A. Rasheed, Rezheen F. Abdulrahman, Nawfal R. Hussein

**Affiliations:** a Medical Department, Akre Technical Institute, Duhok Polytechnic University, Duhok, Kurdistan Region, Iraq; b Pathology and Microbiology Department, College of Veterinary Medicine, University of Duhok, Duhok, Kurdistan Region, Iraq; c Department of Medicine, College of Medicine, University of Zakho, Zakho, Kurdistan Region, Iraq

**Keywords:** MRSA, *16S rRNA*, Phylogenetic tree, *vanA*, Syrian refugees, Duhok

## Abstract

**Objectives:**

The aim of the study was to investigate the genetic relatedness between methicillin-resistant *Staphylococcus aureus* (MRSA) strains isolated from the host community of Duhok City and Syrian refugees based on the partial sequences of the *16S rRNA* gene, and to investigate the prevalence of SCC*mec* and vancomycin resistance.

**Methods:**

Thirty MRSA isolates that had previously been detected and characterized were included. PCR was used for SCC*mec* typing, vancomycin detection, and amplification of the *16S rRNA* gene for sequencing.

**Results:**

The MRSA isolates were identical and highly similar to globally published *S. aureus* strains, especially human nasal cavity strains. The phylogenetic tree showed that the isolates were grouped into two major groups: groups 1 and 2. Group 2 was divergent from group 1 and included isolates from the host community and Syrian refugees (new arrivals). Group 1 included isolates from the three groups, and these were genetically closely related. Most strains in both study groups were SCC*mec* type IVa and clustered on the same lineage of the tree. The highest rate of vancomycin resistance was found among the isolates from refugees.

**Conclusions:**

The close relationship between MRSA strains in the two study groups could be due to the geographical location of neighboring countries, which enhances the spread of MRSA strains between them.

## Highlights

• Methicillin-resistant *Staphylococcus aureus* (MRSA) isolates were identical and highly similar to globally published isolates.
• MRSA strains in the Iraqi and Syrian populations were genetically closely related.
• Study isolates were grouped into two major groups on the phylogenetic tree.
• Most strains in the study were SCC*mec* type IVa, clustered on the same tree lineage.
• The highest rate of vancomycin resistance was found among the isolates from refugees.

## 1 Introduction

*Staphylococcus aureus* is a gram-positive commensal and ubiquitous pathogen that colonizes different parts of the human body, including the skin, nares, and mucosal surfaces (Hanning et al., 2012; Kluytmans and Wertheim, 2005). Approximately one-third of healthy people are colonized by *S. aureus* (Mirkarimi et al., 2016). As an opportunistic pathogen, *S. aureus* is characterized by its ability to acquire and express numerous virulence factors and antimicrobial resistance determinants, both in the hospital and in the community (Witt et al., 2010). Therefore, it can cause a variety of nosocomial and community-acquired infections in humans worldwide, ranging from mild to life-threatening diseases (Al-Talib et al., 2011; Haag et al., 2019; Feng et al., 2008; Song et al., 2013; Turner et al., 2019; Yamada et al., 2011).

Methicillin-resistant *S. aureus* (MRSA) is a major global threat to public health and is a leading cause of skin, soft tissue, bone and joint infections, bacteremia, and endocarditis in both the health care sector and community (Turner et al., 2019). Methicillin resistance is mediated by the acquisition of the *mecA* gene, which encodes a penicillin-binding protein (PBP). The *mecA* gene is localized within the mobile genetic element known as the staphylococcal cassette chromosome (SCC*mec*) (Katayama et al., 2000; Pinho et al., 2001). MRSA strains are resistant to all β-lactam antibiotics and are generated from methicillin-susceptible *S. aureus* (MSSA) (Feng et al., 2008). Vancomycin became and still is one of the therapeutic agents in clinical use for the treatment of *S. aureus* infections, most commonly MRSA infections (Cong et al., 2020). However, vancomycin-resistant *S. aureus* (VRSA) has emerged in recent years, and the resistance is mediated by the *vanA* gene cluster, which is transferred from vancomycin-resistant *Enterococcus* (Cong et al., 2020).

Comparative genomics analysis, including sequence comparison, transcriptome, and proteome levels, has been used increasingly to better understand the pathogenesis, epidemiology, and drug resistance of *S. aureus* (Feng et al., 2008). A variety of molecular techniques have been developed for typing *S. aureus* isolates in order to examine the genetic relatedness and relationships of *S. aureus* strains and provide an insight into the evolution of *S. aureus*. These techniques include multilocus sequence typing (MLST) (Lamers et al., 2012; Maiden, 2006), SCC*mec* typing (Kondo et al., 2005), X region encoding protein A (*spa*) typing (Khademi et al., 2016; Strommenger et al., 2008), *16S rRNA* gene sequence analysis (Takahashi and Kikuchi, 1999), and *agr*-grouping (Feng et al., 2008; Shopsin et al., 2003).

In previous studies, nasal carriage of MRSA isolates was investigated among the host community of Duhok City and Syrian refugees, including both residents in camps and new arrivals at the border (Rasheed and Hussein, 2020a; Rasheed and Hussein, 2020b; Rasheed and Hussein, 2020c; Rasheed and Hussein, 2020d). However, it appears that no study has yet been conducted to examine the phylogenetic analysis of MRSA isolates in Iraq or their phylogenetic relatedness to MRSA from Syrian refugees (refugee residents in camps and new arrivals at the border) based on the *16S rRNA* gene. Therefore, the aim of the present study was to investigate the variation and phylogenetic relationships between MRSA isolates from the aforementioned groups based on partial sequences of the *16S rRNA* gene. Furthermore, the vancomycin resistance among the MRSA isolates was investigated.

## 2 Materials and methods

### 2.1 Bacterial isolates

Thirty MRSA isolates were selected for this study. The isolates were recovered from the nostrils of healthy participants in three different groups: the host community of Duhok City, Iraq (*n* = 10), Syrian refugee residents in camps (*n* = 10), and Syrian refugees upon their arrival at the border (new arrivals) (*n* = 10) (Table 1). The MRSA strains selected for this study were characterized based on phenotypic and genotypic characteristics in previous studies (Rasheed and Hussein, 2020a; Rasheed and Hussein, 2020b; Rasheed and Hussein, 2020c; Rasheed and Hussein, 2020d).

**Table 1:** Methicillin-resistant *Staphylococcus aureus* SCC*mec* typing and vancomycin resistance gene detection for each strain

Isolate	Isolate group^a^	Isolate designation^b^	SCC*mec* type	*vanA* gene	Accession No.
S1	G1	NAR1	IVa	−	**MW644587**
S2	G1	NAR2	I, IVa	−	**MW644588**
S3	G1	NAR3	I, IVa, IVc	−	**MW644589**
S4	G1	NAR4	I, IVa	−	**MW644590**
S5	G1	NAR5	I, II, IVb, V	−	**MW644591**
S6	G1	ND	II, IVb	+	ND
S7	G1	NAR6	IVa	−	**MW644592**
S8	G1	NAR7	IVa	−	**MW644593**
S9	G1	NAR8	IVa	−	**MW644594**
S10	G1	NAR9	IVa, IVc	−	**MW644595**
S11	G2	NAR10	Untypeable	−	**MW644596**
S12	G2	NAR11	IVa	−	**MW644597**
S13	G2	NAR12	IVa	−	**MW644598**
S14	G2	NAR13	IVa	+	**MW644599**
S15	G2	NAR14	IVa	−	**MW644600**
S16	G2	NAR15	Untypeable	+	**MW644601**
S17	G2	NAR16	IVa	−	**MW644602**
S18	G2	NAR17	Untypeable	−	**MW644603**
S19	G2	NAR18	IVa	+	**MW644604**
S20	G2	NAR19	I	−	**MW644605**
S21	G3	NAR20	Untypeable	+	**MW644606**
S22	G3	NAR21	Untypeable	+	**MW644607**
S23	G3	NAR22	Untypeable	+	**MW644608**
S24	G3	NAR23	IVa	+	**MW644609**
S25	G3	NAR24	IVa	−	**MW644610**
S26	G3	ND	Untypeable	−	ND
S27	G3	NAR25	Untypeable	−	**MW644611**
S28	G3	NAR26	IVa	−	**MW644612**
S29	G3	NAR27	IVa	−	**MW644613**
S30	G3	NAR28	IVb	−	**MW644614**

SCC*mec, staphylococcal cassette* chromosome *mec*; ND, not determined.

a Isolate group: G1, host community group; G2, Syrian refugee residents in camps group; G3, Syrian refugees at the border (new arrivals) group.

b Isolate designation: isolate description for NCBI.

### 2.2 Extraction of genomic DNA

The MRSA isolates were maintained in 50% glycerol stocks and stored at −20°C. For DNA extraction, the isolates from stock cultures were plated onto mannitol salt agar (Neogen Company, UK) and incubated at 37°C for 24 h. DNA was extracted using the PureLink Genomic DNA Mini Kit (Invitrogen, Thermo Fisher Scientific, USA) according to the manufacturer's instructions. The concentration and purity of the DNA were measured using a NanoDrop 2000C spectrophotometer (Thermo Fisher Scientific).

### 2.3 SCC*mec* typing by PCR

SCC*mec* typing was conducted using the primers, PCR reactions, and thermocycling conditions applied in a previous study by Rasheed and Hussein (Rasheed and Hussein, 2020c).

### 2.4 Detection of the vancomycin resistance gene

The primer pair vanAF (AATGTGCGAAAAACCTTGCG) and vanAR (CCGTTTCCTGTATCCGTCC) was used to amplify *vanA* (Lu et al., 2001). Each reaction consisted of a total volume of 20 μl, including 10 μl of Hot Start Master Mix (GeNet Bio, Korea) (Hot Start Prime Taq DNA Polymerase (1 unit/10 µl), 2× reaction buffer, MgCl_2_ (4 mM), enzyme stabilizer, loading dye, and nucleotides (pH 9.0, 0.5 mM each)), 1 μl of each primer at a concentration of 10 pmol/µl, 4 μl of the DNA template, and 4 μl of dH_2_O. The amplification was performed in a GeneAmp PCR System 9700 Thermal Cycler (Applied Biosystems) using the following PCR conditions: initial denaturation of 94°C for 4 min and 35 cycles of 94°C for 1 min, 58°C for 1 min, and 72°C for 1 min, followed by a final extension of 72°C for 6 min. The PCR products were visualized on 2% agarose gel with Prime Safe Dye (GeNet Bio, Korea) at 80 V for 1 h.

### 2.5 Amplification of *16S rRNA*

The partial sequence of *16S rRNA* was amplified using the primer pair 27F (AGAGTTTGATCMTGGCTCAG) and 1492R (TACGGYTACCTTGTTACGACTT) according to Rohwer et al. (Rohwer et al., 2001). A total reaction volume of 30 µl was used for the reaction. Each reaction consisted of 15 µl of Hot Start Master Mix (GeNet Bio, Korea), 1 µl of each primer (10 pmol/µl), 2 µl of template DNA, and 11 µl dH_2_O. Amplification was performed in a GeneAmp PCR System 9700 Thermal Cycler (Applied Biosystems) with the following conditions: initial denaturation at 94°C for 4 min, followed by 30 cycles of denaturation at 94°C for 30 s, annealing at 57°C for 45 s, and extension at 72°C for 90 s, and a final extension at 72°C for 7 min. The size of the amplified products was estimated using 1% (w/v) agarose gel electrophoresis and visualized under UV light.

### 2.6 *16S rRNA* gene sequencing

For purification of the *16S rRNA* gene before sequencing, the PrimePrep Gel Purification Kit (GeNet Bio) was used to purify DNA from agarose gel according to the manufacturer's instructions. Finally, 40 µl of purified products was sequenced using the Sanger sequencing technique by the Humanizing Genomics Macrogen sequencing service (Seoul, South Korea).

### 2.7 Analysis of sequences and phylogenetic tree

BioEdit sequence alignment editor (version 7) was used to view and analyze the sequence files (Hall, 1999). Sequence alignments and comparisons were also conducted using the BioEdit software. The identities of the sequenced *16S rRNA* of *S. aureus* obtained in this study with reference sequences published in the GenBank database were obtained using Basic Local Alignment Search Tool (BLAST) analysis (https://www.ncbi.nlm.nih.gov/). Multiple sequence alignment with ClustalW was performed using Molecular Evolutionary Genetics Analysis (MEGA X) software (Kumar et al., 2018). A neighbor-joining phylogenetic tree was constructed using MEGA X based on the Jukes–Canter correction model and bootstrap method (1000 replications).

## 3 Results

### 3.1 SCC*mec* typing

SCC*mec* typing was performed for each strain of MRSA included in this study. Group 1 isolates had the most diverse SCC*mec* types with different bounds. However, the majority of isolates in group 2 had one bound that belonged to the IVa type and three isolates were untypeable. On the other hand, six of the isolates in group 3 belonged to the IVa type and six were untypeable. The results are shown in Table 1.

### 3.2 Vancomycin resistance of MRSA

The *vanA* gene was detected in 26.7% (8/30) of the MRSA isolates. Only one (3.3%) MRSA isolate from the host community was found to be positive for the *vanA* gene. The results also showed that the *vanA* gene specific for vancomycin resistance was found in three (10%) MRSA isolates from the Syrian refugee residents in camps and four (13.3%) MRSA isolates from the Syrian refugees who were new arrivals at the border (Table 1).

### 3.3 Sequence comparison and phylogenetic analysis

**Figure 1:**
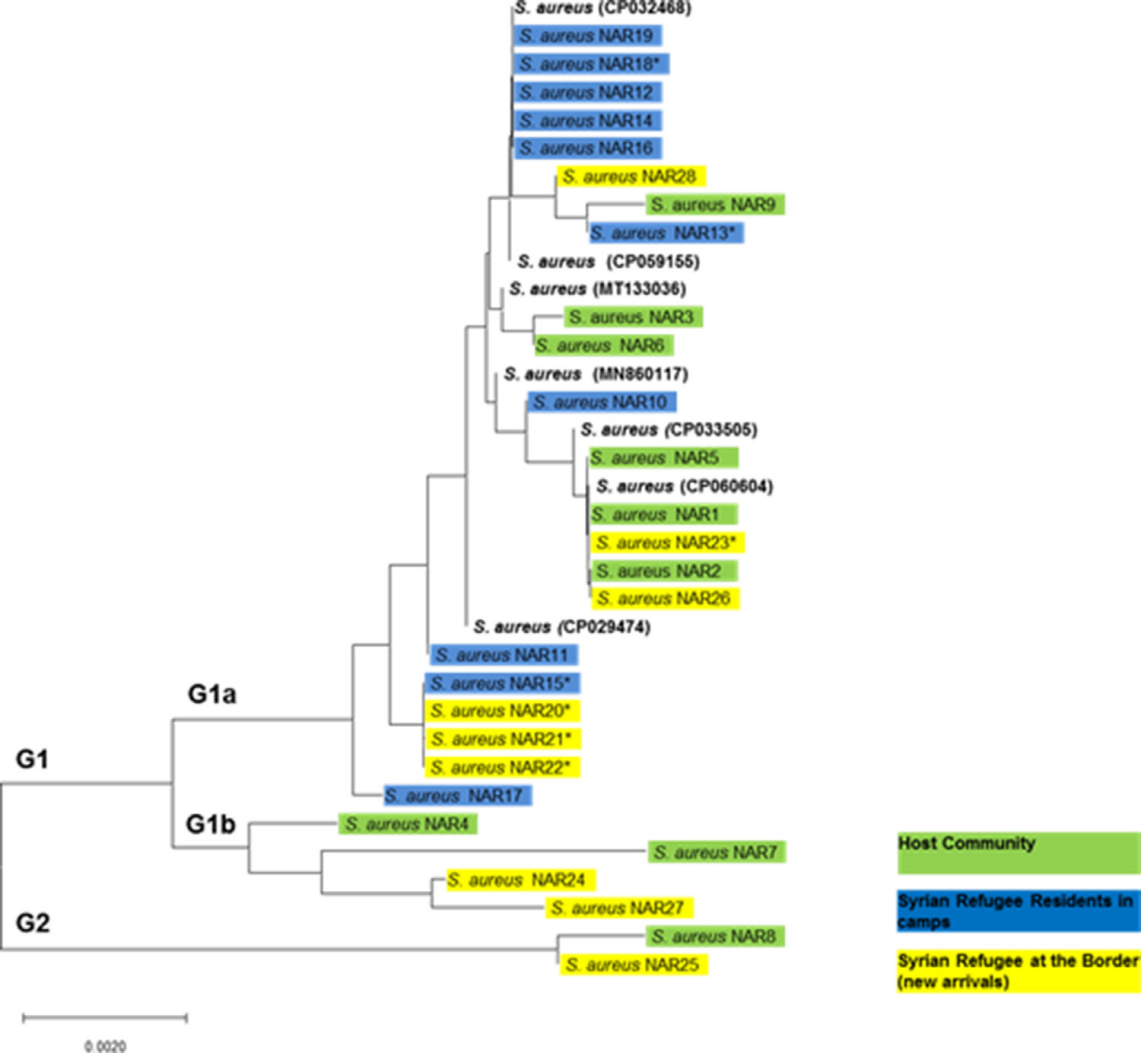
Phylogenetic relatedness of MRSA isolates based on the *16S rRNA* gene sequences. The tree was constructed with MEGA X using the neighbor-joining method and a bootstrap value of 1000 repetitions. The 28 *16S rRNA* gene sequences of the MRSA isolates obtained in the current study were designated as *Staphylococcus aureus* NAR1–NAR28, as shown in the tree, and were deposited in the GenBank database under accessions numbers **MW644587** to **MW644614**. Note: *vanA gene positive.

Three groups of MRSA strains from the three different communities representing different SCC*mec* types were used in this study to analyze the phylogenetic relationship of these strains based on partial sequences of *16S rRNA*. Thirty partial *16S rRNA* sequences were obtained from 30 *S. aureus* isolates. The sequences were cleaned and confirmed as *S. aureus* using BLAST analysis against the globally published NCBI database. However, two sequences, one from the host community and one from Syrian refugees (new arrivals at the border) were excluded from the analysis because the quality of the sequences was poor and therefore they were not included in the analysis (Table 1). Thus, 28 partial sequences of *16S rRNA* were analyzed: nine from the host community, 10 from Syrian refugee residents in camps, and nine from Syrian refugees who were new arrivals at the border. The *16S rRNA* sequences in this study were renamed as *S. aureus* NAR1–NAR28; these were deposited in the GenBank database under accession numbers **MW644587** to **MW644614** (Table 1 and Figure 1).

Multiple sequence alignment of the 28 partial sequences showed that the total nucleotide variation among all sequences was 1.5% (15 polymorphic nucleotide sites) and the amino acid variation was 2.9% (10 variable amino acid sites). Pairwise differences in nucleotide and amino acid sequences were calculated, and the results showed that the pairwise differences ranged from 0 to 8 nucleotide sites and 0 to 5 amino acid positions. Nucleotide sequence comparison of *16S rRNA* sequences using BLAST analysis against globally published *S. aureus* sequences showed similarity with *S. aureus* from different hosts, isolation sites, and clinical samples. The similarity ranged from 97% to 100%. The MRSA isolates used in this study were isolated from the nostrils of healthy participants from the host community and healthy Syrian refugees. Thus, in order to construct a phylogenetic tree, *16S rRNA* genes from human nasal carriage from different countries were obtained from GenBank and included within the phylogenetic tree. The comparison was performed with the following *S. aureus* reference sequences (GenBank accession numbers): **CP032468,CP059155,MT133036,MN860117,CP033505,CP060604**, and **CP029474** (Figure 1). They were approximately 97% to 100% similar to the isolates used in the current study.

**Figure 2:**
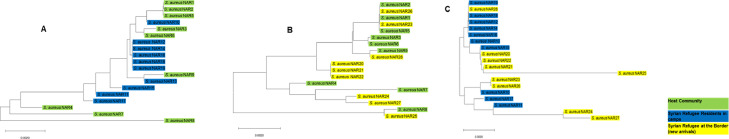
Phylogenetic relatedness of MRSA isolates based on the *16S rRNA* gene sequences: (A) host community and Syrian refugee residents in camps; (B) host community and Syrian refugees (new arrivals) at the border; (C) Syrian refugee residents in camps and new arrivals. The tree was constructed with MEGA X using the neighbor-joining method and a bootstrap value of 1000 repetitions.

The *16S rRNA* phylogenetic tree showed that the 28 sequences (Figure 1) were grouped into two major groups (G1 and G2). The majority of the isolates from the host community and Syrian refugees (26 of 28) were clustered within G1, which was divided into two subgroups: G1a and G1b. However, a low degree of both nucleotide and amino acid variation was found among the isolates within each subgroup. Although the isolates within G1a (4 of 26) were grouped within G2, they were different from G1a. G2 represents only two isolates, including one isolate from the host community and one from Syrian refugees upon their arrival at the border, and both were divergent from the isolates in G1. G1 represents 26 isolates from the host community and Syrian refugees (both residents and new arrivals at the border) with a variation of only 11 polymorphic nucleotide sites and nine amino acid positions. The isolates were very closely related, with pairwise differences of 0 to 7 and 0 to 5 nucleotides and amino acids, respectively. The overall results showed that the isolates from both the Iraqi community and Syrian refugees were genetically similar and closely related, as shown in Figure 2.

## 4 Discussion

*Staphylococcus aureus* strains are common commensals of the upper respiratory tract distributed in both communities and hospitals, causing serious infections worldwide. The prevalence of MRSA nasal carriage in the community of Duhok City is significant, as reported previously, and the virulence determinants have also been determined (Assafi et al., 2015; Habeeb et al., 2014; Hussein et al., 2015; Hussein, 2016; Hussein et al., 2019; Rasheed and Hussein, 2020a). Limited data are available regarding the prevalence of MRSA among Syrian refugees in Iraq. Consequently, for the first time in Iraq, MRSA isolates from Syrian refugees were characterized in detail by Rasheed and Hussein (Rasheed and Hussein, 2020a; Rasheed and Hussein, 2020b; Rasheed and Hussein, 2020c; Rasheed and Hussein, 2020d). However, until now, no study has been conducted to investigate the phylogenetic relatedness of MRSA strains isolated from both the host community and Syrian refugees in Iraq. Therefore, the aim of the present study was to investigate their relatedness based on the sequence alignment of 28 partial sequences of the *16S rRNA* gene obtained from MRSA among the host community and Syrian refugees (residents and new arrivals at the border).

The results of this study showed that MRSA isolates from both communities were closely related and were genetically similar. SCC*mec* types were characterized among the MRSA strains examined in this study. Furthermore, characterization of the SCC*mec* groups of the same MRSA isolates among both communities showed that the host community and Syrian refugees shared the same SCC*mec* types, including SCC*mec* type IVa; in addition, several strains from the Syrian refugees were untypeable. The tree showed that the strains with the untypeable SCC*mec* type were identical and clustered together. Moreover, most of the isolates with SCC*mec* type IVa clustered within the same lineages, as shown in Table 1 and Figure 1. These findings confirmed that the MRSA strains in this study were genetically related.

MRSA strains are considered a threat to public health in both the community and hospitals because of the emergence multidrug-resistant strains (Shajari et al., 2017). Vancomycin is the drug of choice for the treatment of MRSA infections. However, complete resistance to vancomycin has emerged and has become a serious public health concern in different countries (Askari et al., 2015; Cesur et al., 2012; Shajari et al., 2017). In this study only one isolate showed resistance to vancomycin. However, in previous studies performed in Duhok City, no isolates showed resistance to vancomycin (Assafi et al., 2015; Habeeb et al., 2014; Hussein et al., 2015). The presence of one VRSA isolate in the host community could be an alert for the emergence of VRSA. The results showed the highest number of *vanA*-positive isolates were among the Syrian refugees, which can be considered a risk for the spread of these isolates into the host community. The results confirm the importance of implementing infection control measures among refugees and communities to prevent the spread of vancomycin-resistant MRSA isolates. Studies on the detection of VRSA among Syrian refugees in other countries are scarce. In one study from Syria, VRSA isolates were found in 2% of the isolates recovered from 202 volunteer medical staff in major hospitals in three provinces using susceptibility testing, suggesting the presence of VRSA in Syria (Tabana et al., 2015).

Although the *16S rRNA* gene is the most common gene studied in phylogenetic studies, the use of this method is very limited because of the high sequence similarity among the different staphylococcal species (Taponen et al., 2006). Thus, additional genes have recently been used to describe the phylogenetic relationships and closely related species (Lamers et al., 2012). No data or information was found regarding the phylogenetic relatedness of MRSA strains recovered from the host community and refugees in other studies.

In conclusion, the findings of this study showed a clear genetic relationship between MRSA strains in the Iraqi and Syrian communities and that they were closely related. This close relationship may be due to the geographical location of neighboring countries, which enhances the spread of MRSA strains between them. The presence of VRSA among the Syrian refugees indicates the risk of the spread of these isolates into the host community. Strict control measures should be applied among refugees and those in the host community in order to prevent the spread of VRSA within both communities.

## Author contributions

NAR conducted the sample collection, laboratory work, data analysis, and the manuscript writing. RFA worked on the sequence analysis, phylogenetic tree construction, and writing the manuscript. NRH contributed to the manuscript editing, reviewing, and supervising the work. All authors read and approved the final manuscript.

## Declarations

*Funding:* This research did not receive any specific grant from funding agencies in the public, commercial, or not-for-profit sectors.

*Ethical approval:* Consent was obtained from all participants for the use of their samples for research purposes and signed consent was received from them prior to sample collection. Ethical approval for the study was given by the Scientific Committee of the College of Medicine, University of Zakho, Kurdistan region of Iraq.

*Conflict of interest:* There is no conflict of interest regarding the publication of this article.
